# Proteomics for the Investigation of Mycobacteria

**Published:** 2017

**Authors:** J. A. Bespyatykh, E. A. Shitikov, E. N. Ilina

**Affiliations:** Federal Research and Clinical Center of Physical-Chemical Medicine of Federal Medical Biological Agency, Malaya Pirogovskaya str. 1a, Moscow, 119435, Russia

**Keywords:** Mycobacterium tuberculosis, proteins, proteome, proteomics, tuberculosis

## Abstract

The physiology of *Mycobacterium tuberculosis*, the causative agent of tuberculosis, is being studied with intensity. However, despite the genomic and transcriptomic data available today, the pathogenic potential of these bacteria remains poorly understood. Therefore, proteomic approaches seem relevant in studying mycobacteria. This review covers the main stages in the proteomic analysis methods used to study mycobacteria. The main achievements in the area of *M. tuberculosis *proteomics are described in general. Special attention is paid to the proteomic features of the Beijing family, which is widespread in Russia. Considering that the proteome is a set of all the proteins in the cell, post-translational modifications of mycobacterium proteins are also described.

## INTRODUCTION

The systems biology of prokaryotes seeks to understand how the physical and chemical properties and the nature of the interaction between biomolecules are related to the formation of the phenotypic properties of microorganisms. Nowadays, the nucleotide sequence of a prokaryotic genome can be deciphered within hours. Nevertheless, despite the fact that the genome encodes, either directly or indirectly, key cell biomolecules such as RNA and proteins, it remains impossible to characterize their functional properties based on information regarding the genomic sequence. Accurate and reproducible methods for the quantification of all components under various conditions are needed to study the structure, function, and molecular mechanisms of regulation in these molecular systems. To date, these assessments have become common for RNA [1-4]. However, they still lag behind in sensitivity and representativeness due to technical limitations at the protein level.

Clinically relevant microorganisms, in particular mycobacteria, have been studied most intensely using systems biology methods. To date, 213 mycobacterial species have been described, many of which are associated with infectious processes in humans or animals [5]. These species include *Mycobacterium tuberculosis*, *M. leprae*, and *M. ulcerans*, which cause tuberculosis, leprosy, and Buruli ulcer, respectively. According to international statistics, approximately one-third of the world’s population is infected with tuberculosis; approximately 1.3 million deaths from the disease were registered in 2015 [6]. Not surprisingly, the features of the physiology and molecular organization of *M. tuberculosis *are of the greatest interest.

Until recently, major efforts have focused on the features of the genomic organization of the tuberculosis pathogen. Today, genomic sequencing data for more than 10,000 *M. tuberculosis *strains with different phenotypes and genotypes are available. However, the experience of applying the whole genome sequencing technology with subsequent comparative analysis reveals the limited applicability of the approach for a complete description of the causes behind drug resistance and pathogenicity [7]. Thus, the majority of the point mutations that distinguish groups of strains have been found in the promoter regions of the genes and/ or regions encoding proteins with a hypothetical function and playing an unknown role in the physiology of mycobacteria. In this context, a functional analysis of the information enciphered in the pathogen genome performed using proteomic testing, including quantitative proteomics, becomes relevant.

It is worth noting that isolation of DNA and RNA from mycobacteria and further manipulations have been described in a large number of protocols [8-10] which are applied in various laboratories [11-16]. The situation with protein isolation, especially isolation of the total protein fraction required to obtain the proteome, is quite the opposite. The features of organization of the cell wall, which is resistant to environmental factors, acids and alkalis, make *M. tuberculosis *a rather complex target for a proteomic analysis. This, in its turn, requires the development of unique conditions for protein extraction. The implemented protocols of proteomic analysis of *M. tuberculosis *should also be sufficiently effective, taking into account the complexity of accumulation of a large bacterial mass due to the extremely slow culture growth.

This review looks into the development of tools for the proteomic analysis of mycobacteria in the chronological aspect of increasing their informative value and the accuracy of assessments.

## PROTEOMIC APPROACHES FOR MYCOBACTERIAL STUDIES

**Development of the methods of proteomic analysis of mycobacteria**

Mark Wilkins was the first to introduce the term “proteome” in 1986, which combined two words: “PROTEin” and “genOME” [17]. Proteome is the set of all proteins in the cell, including the changes that occur over time or under some factors. In 1997, the term “proteomics” appeared by analogy with genomics, which studies genes and their functions [18].

Proteomics studies the set of proteins synthesized by an organism/cell in a specific environment and at a particular stage of the cell cycle. It describes the qualitative composition of proteins, their relative representation, interaction with other macromolecules, and post-translational modifications (PTM) [19-21].

Proteomics still lags behind genomics and transcriptomics due to instrumental problems and the insufficient sensitivity of the existing methods. However, the number of works that utilize proteomic methods for studying infectious agents is on the increase.

**Table 1:** The major studies that have contributed to the development of mycobacterial proteomics

Year	Title	Number of identified proteins	Reference
1997	Definition of Mycobacterium tuberculosis culture filtrate proteins by two-dimensional polyacrylamide gel electrophoresis, N-terminal amino acid sequencing, and electrospray mass spectrometry	32	M.G. Sonnenberg and J.T. Belisle [26]
1999	Comparative proteome analysis of Mycobacterium tuberculosis and Mycobacterium bovis BCG strains: toward functional genomics of microbial pathogens	107	P.R. Jungblut et al. [27]
2000	Toward the proteome of Mycobacterium tuberculosis	167	I. Rosenkrands et al. [24]
2003	Comprehensive proteomic profiling of the membrane constituents of a Mycobacterium tuberculosis strain	739	S. Gu et al. [28]
2004	Complementary analysis of the Mycobacterium tuberculosis proteome by two-dimensional electrophoresis and isotope-coded affinity tag technology	361	F. Schmidt et al. [29]
2005	Mycobacterium tuberculosis functional network analysis by global subcellular protein profiling	1044	K.G. Mawuenyega et al. [30]
2010	Using a label-free proteomics method to identify differentially abundant proteins in closely related hypo- and hypervirulent clinical Mycobacterium tuberculosis Beijing isolates	1668	G.A. de Souza et al. [31]
2011	Comparison of membrane proteins of Mycobacterium tuberculosis H37Rv and H37Ra strains	1578	H. Malen et al. [32]
2011	Characterization of the Mycobacterium tuberculosis proteome by liquid chromatography mass spectrometry-based proteomics techniques: a comprehensive resource for tuberculosis research	1051	C. Bell et al. [33]
2011	Proteogenomic analysis of Mycobacterium tuberculosis by high resolution mass spectrometry	3176	D.S. Kelkar et al. [34]
2013	The Mtb Proteome Library: A resource of assays to quantify the complete proteome of Mycobacterium tuberculosis	3894	O.T. Schubert et al. [23]
2014	Disclosure of selective advantages in the “modern” sublineage of the Mycobacterium tuberculosis Beijing genotype family by quantitative proteomics.	2392	J. de Keijzer et al. [35]
2015	Quantitative proteomic analysis of M. tuberculosis cluster Beijing B0/W148 strains	1868	J. Bespyatykh et al. [36, 37]

R. Aebersold et al. made the most significant contribution to the development of the proteomics of mycobacteria [22, 23]. The main studies on mycobacterial proteins are presented in *table*.

**Fig. 1:**
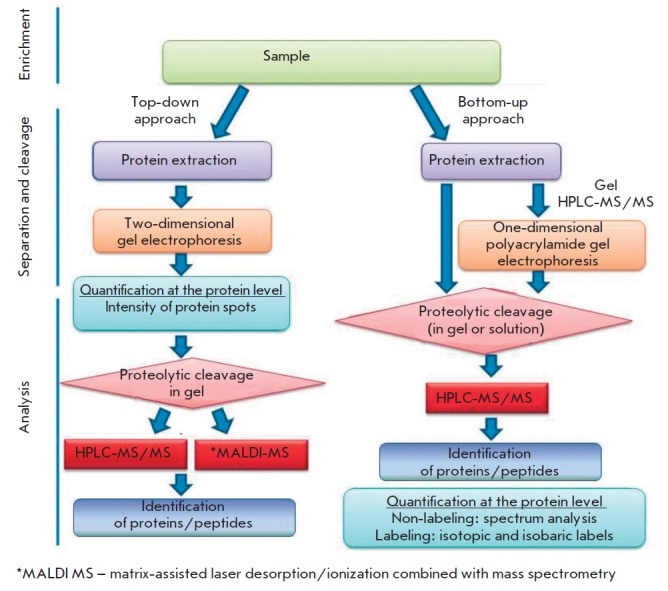
Main proteomic approaches: top-down and bottom-up. Adapted from [118] with modifications.

The early studies in the field of *M. tuberculosis *proteomics conducted at the end of the 20^th^ century relied on the strategy of the so-called top-down proteomics, the main principle of which is sorting the intact proteins isolated from a biological sample based on their physical and chemical properties (using gel electrophoresis and gel filtration) and subsequently identifying them using mass spectrometric (MS) methods (*Fig. 1*). This approach enabled the identification and quantification of about 100 mycobacterial proteins [24], which does not exceed 3% of the total proteome of *M. tuberculosis*.

**Fig. 2:**
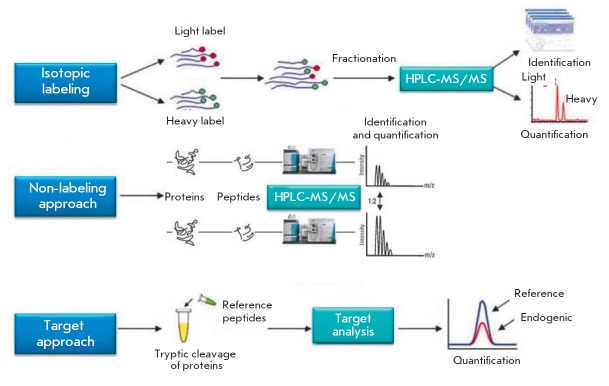
Methods of proteomic analysis implementing the bottom-up approaches

Further development of the tools of a proteomic analysis opened up new ways for exploring tuberculosis and facilitated the study of many complex issues, including the interactions between a bacterium and the host cell. The technologies that implement the strategy of the so-called bottom-up proteomics are considered to be most effective for obtaining the total proteome [20, 21]. These technologies are based on the fact that the total set of proteins isolated from a biological object is first proteolytically cleaved into peptides, which are then continuously analyzed by high-performance liquid chromatography coupled with tandem mass spectrometry (HPLC-MS/MS) (*Fig. 1*). The variety of bottom-up methods at the stage of MS experiment can be divided into (1) exploratory or panoramic (shotgun proteomics) and (2) confirming (target) ones (*Fig. 2*). The first ones are aimed at identifying and quantifying as many proteins as possible and allow one to identify up to 1,000 *M. tuberculosis *proteins in a single MS run [25]. The latter methods are developed for tracking a relatively small set of proteins/peptides, which is defined prior to an experiment, in numerous samples with the highest sensitivity, accuracy, reproducibility and capacity available for the method (e.g., multiple reaction monitoring, MRM).

The most accurate results in a quantitative comparison of samples using the panoramic approach are achieved by isotopic labeling of one of the analytes [38]. In particular, stable isotope labeling by amino acids in a cell culture (SILAC) is based on the incorporation of essential amino acids containing stable isotopes (usually ^13^C/^15^N arginine and/or lysine) in the protein structure [39, 40]. It is assumed that the analyzed cells do not synthesize lysine and arginine but utilize only the labeled amino acids present in the medium. However, *M. tuberculosis *is capable of endogenously synthesizing lysine, which immediately limits the possibilities of this approach. The main emphasis in the quantitative proteomics of mycobacteria has since recently been placed on using non-labeling methods for the MS quantification of proteins that are characterized by greater sensitivity and performance [31, 41].

New approaches to the accumulation and processing of mass spectra, e.g., SWATH™ designed by ABSciEX, combine the panoramic (data-independent acquisition, DIA) and confirming (data-dependent acquisition, DDA) techniques aimed at minimizing the limitations of each one of them [42]. Moreover, SWATH™ does not require the selection of individual parent ions, while precursor ions are skipped by large windows (e.g., of about 25 Da). Thus, SWATH™ allows one to identify and quantify a large set of proteins, similarly to the conventional panoramic approach but with a precision and reproducibility typical of that of MMP for a larger number of samples.

## PROTEOMIC FEATURES OF CERTAIN MYCOBACTERIAL GROUPS

**Proteomic characterization of the *M. tuberculosis* H37Rv strain**

*M. tuberculosis *H37Rv is now the most well-studied mycobacterial strain. The genomic sequence of this strain was completely deciphered in 1998 [43]. It is not surprising that it is also the proteome of this strain that has been studied as thoroughly as possible. The existence of 97% out of 4,012 annotated proteins has been confirmed by genomic sequences using comprehensive proteomic approaches [23]. Pools of cell wall and membrane proteins [32, 44], cytosolic proteins [25, 30, 45], and secreted proteins detected in a culture filtrate [46] have been described.

An analysis of the proteins of DosR regulon, which is associated with the anaerobic survival of *M. tuberculosis*, revealed changes in their representation in a strain H37Rv bacterial culture under hypoxic conditions [29]. In particular, representation of the HspX protein increased 340-fold during hypoxia compared to that in the culture under normal conditions. It is worth noting that this regulon had earlier been studied only at the level of transcripts [47, 48].

Of special interest are the studies focused on a simulation of the infectious process and assessment of the protein profile of *M. tuberculosis *under conditions maximally similar to the existence of bacterial cells in a living organism. Cho et al. [49] conducted a comparative proteomic analysis of the proteins of a latent H37Rv strain at the exponential, logarithmic, and stationary growth phases using the technique of site-specific labeling of cysteine residues (isotope-coded affinity tags, ICAT) based on covalent labeling of the cysteine residue in the polypeptide chain by chemically identical but isotopically different reagents [22, 29]. The results allowed them to identify highly enriched proteins typical of the exponential and stationary phases: 193 and 241 proteins, respectively. Most of these systems were associated with the pathways of protein degradation and energetic metabolism.

The differences in the proteomic profiles of a virulent H37Rv strain and avirulent mycobacteria (*M. tuberculosis *H37Ra, *M. bovis *BCG) were evaluated in order to study the virulence factors of *M. tuberculosis *and to identify potential candidates for designing vaccines. A similar representation of the majority of membrane proteins was found in the strains H37Rv and H37Ra, while the representation of 121 proteins in these strains varied more than fivefold. Further research into membrane lipoproteins and data on their regulation suggested that the change in the metabolic state might play some role in the increased virulence [32]. The study of Esat-6 proteins and ESAT-6-like proteins, which are found in H37Rv strains but not in the H37Ra strain, showed mutations in the genes of five ESAT-6-like proteins in the strain H37Ra. It is worth mentioning that the 6 kDa antigen (Esat-6) forms a heterodimeric complex with the CFP-10 protein [50]. Therefore, the ESAT-6/CFP-10 system is believed to be associated with *M. tuberculosis *and inhibit the fusion of phagosome and lysosome in host macrophages, thereby preventing the destruction of mycobacterial cells [51].

Although H37Rv and *M. bovis *BCG share more than 99.9% homology at the genomic level, 294 proteins that differ statistically significantly between the two strains have been identified [41]. A lack in certain regions of difference (RD) was previously estalished in the BCG genome using a comparative genomic analysis of these strains; the lack of pathogenicity was associated with a loss of the corresponding genes [52]. Hence, a series of different proteins corresponded to the described RDs [41, 53, 54]. Among them, special attention was paid to the ESAT-6 system, the proteins of which had been previously suggested as candidates for designing a new vaccine [55]. In addition, 22 differentially expressed proteins, such as acetyl-CoA acetyltransferase (Rv0243) and several Esat-6-like proteins (Rv1198, Rv1793), were proposed for designing diagnostic and vaccine agents [54].

**Proteomic characterization of strains of the Beijing *M. tuberculosis* family**

As noted above, most studies have focused on the proteome of the laboratory *M. tuberculosis *H37Rv strain, while data on the proteomic characteristics of other genetic families is very scarce.

According to the most elaborate classification, *M. tuberculosis *strains are divided into seven genetic lines [56]. From a clinical point of view, the Beijing family is of undoubted interest. The strains of this family belong to the phylogenetic line 2 and are represented in the largest number of countries globally: 13% of the global amount of isolates [57]. In addition, they are characterized by an association with the development of drug resistance [58] and greater virulence compared to other families [59].

A comparison of the proteomes of the Beijing and H37Rv strains showed significant differences between them. Representation of the proteins Rv0129c, Rv0831c, Rv1096, Rv3117, and Rv3804c, which belong to known virulence factors [60], was higher in the Beijing strains than in H37Rv. Meanwhile, the content of proteins Hsp65 (Rv0440), Pst1 (Rv0934), and Rv1886c, which are basic antigens whose reduced production may contribute to the avoidance of the host’s immune response by mycobacteria [61, 62], was decreased. Furthermore, proteins of the efflux pumps Rv0341, Rv2688c, and Rv3728 were found only in the Beijing strains [35].

However, we found only two papers [31, 35] that focused on the variety of proteins in the Beijing strains. In the first study, de Souza et al. compared the proteomes of the hypo- and hypervirulent strains of the family and described about 50 proteins that are highly represented in each group, while a total of 1,668 proteins have been identified [31]. Representation of the ESAT-6 protein was shown to be lower in hypervirulent strains than that in hyporvirulent strains. Moreover, this result was additionally confirmed by a comparative assessment of the expression of the corresponding gene at the transcriptional level. The increase in the relative representation of the ESAT-6 protein was previously regarded as a characteristic of virulent strains [51, 55]. This ambiguous result proves that the role of ESAT-6 secretion pathways in the pathogenicity of *M. tuberculosis *is rather complex. This can be an additional argument for the phenotypic differences between the strains of the Beijing and H37Rv families.

Another study by de Keijzer et al. was devoted to comparing the proteins of the *M. tuberculosis *strains that belong to ancient (atypical) and modern (typical) sublineages of the Beijing family [35]. Isotope labeling of amino acids in cell culture combined with HPLC-MS/MS enabled the identification and quantification of 2,392 proteins. Despite the fact that the protein profiles of both sublineages turned out to be very similar, differences in the representation of four proteins were found: MmpL4 (Rv0450c), Rv3137, Rv1269c, and SseA (Rv3283). Among these proteins, the representation of MmpL4 (Rv0450c) and Rv3137 in the group of typical strains was significantly higher than that in the atypical ones. The SseA (Rv3283) protein is among the underrepresented proteins of the modern Beijing family; its transcriptional level was also reduced.

It is worth noting that Beijing family strains hold a dominant position (50–80%) in the population structure of the tuberculosis pathogen in Russia [63, 64]. The members of this family can be divided into several types based on a VNTR analysis [65, 66]. Types M2 and M11 are most widespread in Russia and comprise about 80% of all detected isolates [66, 67]. After exploring Beijing strains B0/W148 belonging to the M11 type, we confirmed their association with the development of multidrug resistance, found new potential ways of formation of anti-TB drug resistance, and described their unique genomic rearrangement [15].

In turn, we performed a comparative proteomic analysis of Beijing B0/W148 cluster strains and the H37Rv strain [37]. A total of 1,868 proteins of B0/W148 cluster strains and 1,560 proteins of the strain H37Rv have been identified. Among them, a group of 266 differentially represented proteins was isolated. The representation of 41 proteins in Beijing B0/W148 cluster strains was higher than in strain H37Rv, while the representation of 225 proteins was lower. We evaluated the potential biological effect of these differences on the basis of an enrichment of the functional categories of the proteins during a Gene Ontology (GO) analysis and recruitment of the gene regulatory network [68]. We assumed that some of the aforementioned features of the B0/W148 cluster representatives contribute to increased virulence and successful dissemination of these strains. In particular, we observed an increased representation of the enzymes responsible for the biosynthesis of long-chain fatty acids along with reduced representation of the proteins responsible for their degradation. Mycobacteria utilize long-chain fatty acids to obtain mycothiol acids and various lipids, which are considered to be the major virulence factors of *M. tuberculosis *manifesting themselves at the initial stages of infection when bacteria penetrate a macrophage. We have also noted an increase in the representation of the HsaA protein involved in the degradation of steroids. *M. tuberculosis *was shown to utilize extracellular cholesterol as a source of energy and to biosynthesize cell wall lipids. These observations may argue for the increased survival of mycobacteria in macrophages, which is a known characteristic of Beijing B0/W148 cluster strains [69, 70]. In addition, we found a very low level of the SseA protein in B0/W148 strains, which may possibly lead to the accumulation of reactive oxygen species and, as a result, DNA damage. This, in turn, can yield a wide spectrum of genetic variants that contribute to the survival of the bacterial cell under selection, in particular during drug therapy.

Studies in the field of proteomics of drug-resistant *M. tuberculosis *strains are also worth our attention [71-73]. For example, a comparison of the resistant and susceptible strains revealed five proteins (Rv0491, Rv1446c, Rv2145, Rv2971, and Rv3028c) with increased representation in isoniazid-resistant strains [72]. These are membrane proteins that can potentially serve as targets for new therapeutic agents. An analysis of the aminoglycoside-resistant strains revealed an increased representation of the proteins Rv0685, Rv1876, and Rv3841, which are associated with iron metabolism [73]. Assimilation and utilization of iron play an important role in the growth, virulence, and formation of latent *M. tuberculosis *species. Pandey and Rodriguez have suggested that ferritin (Rv3841) is required for maintaining iron homeostasis in mycobacterial cells, while its lack renders bacteria more susceptible to antibiotics [74]. Increased representation of the Rv1876 and Rv3224 proteins involved in iron metabolism was also mentioned in a similar study, and their possible role in the development of resistance to second-line anti-TB drugs was suggested [71]. A comprehensive comparison of sensitive strains and multidrug-resistant strains revealed such virulence factors in resistant strains as catalase/peroxidase (Rv1908c), which is activated in phagosomes [50]. It was demonstrated previously that catalase/peroxidase activity is required for cell growth and persistence in mice, guinea pigs [75], and human peripheral blood monocytes [76]. In addition, the proteins Rv0036, Rv2032c, Rv0635, Rv1827, and Rv2896c, which partake in cellular metabolism and contribute to intracellular survival, have been identified. In one of the recent studies, the proteins Rv2031c, Rv3692, and Rv0444c were suggested for use as biomarkers for effective serodiagnosis of resistant mycobacterium strains [77].

**Analysis of post-translational modifications**

The advance in proteomic analysis techniques makes it possible to draw up an inventory of proteins, compare their representation, and identify the known post-translational modifications (PTM).

Today, PTMs of *M. tuberculosis *proteins such as *O*-glycosylation [33, 78-84], phosphorylation [85, 86], methylation [87], acetylation [88], lipidation [33, 81, 82, 89-93], deamidation [94], N-formylation [95, 96], and ubiquitination [94, 97-102] have been described.

In particular, a characterization of the ubiquitin-like protein Rv2111c of *M. tuberculosis *made it the first described ubiquitin-like bacterial system [101]. Ubiquitination (attachment of a several-molecules-long chain of the short protein ubiquitin to the protein) is a universal PTM in eukaryotes, acting as a signal for the protein’s cleavage by proteasome. It is difficult to identify the corresponding proteins, since they are quickly eliminated. Ubiquitination sites were initially found in 41 *M. tuberculosis *proteins [103]. A total of 602 ubiquitin-like mycobacterial proteins have been described to date. However, modification sites have been experimentally identified only in 55 of them [102].

Phosphorylation is another common PTM. A total of 516 sites of serine/threonine phosphorylation by kinases were found in 301 *M. tuberculosis *proteins. This data was used to search for potential motifs to explain phosphorylation by kinases. Remarkably, six out of eight tested kinases contained conserved motifs, thus indicating that there is a high level of redundancy of kinase function in *M. tuberculosis* [86].

The lipoproteins exported through the general secretory pathway and processed by signal peptidase II are modified by acylation of N-terminal cysteine. In mycobacteria, these modifications have not yet been fully characterized. Some lipoproteins can also be *O-*glycosylated near the N-terminus. This region often contains several threonine residues that act as a target for the aforedescribed modifications. The role of these modifications is still unknown. However, there is speculation that they protect proteins from proteolytic cleavage. At least some lipoproteins are exported to the bacterial cell surface [104, 105] using a lipid tail anchored in the outer membrane [106]. Therefore, the N-terminal regions of the polypeptide chain are susceptible to proteolytic cleavage and many lipoproteins, slightly truncated and in the soluble form, can be found in culture supernatants. The glycosylated lipoproteins MPT 83 [107] and SodC [82] are currently the best characterized. *O*-mannosylation (a special type of glycosylation), as shown in a mouse model, reduces the pathogenic potential of *M. tuberculosis *[108]. More than 40 *O*-glycosylated proteins were found in the supernatant of a *M. tuberculosis *culture using proteomic approaches [83, 109]. Only one glycosylated protein not belonging to lipoproteins is known: MPT 32, or Apa [78]. Apa is one of the most common extracellular proteins secreted via the general secretory pathway [110].

Finally, detection of the TB antigen, a surface heparin-binding hemagglutinin that is considered to be a component in the design of a new vaccine, arouses interest [111, 112]. The uniqueness of this protein holds in that several lysine residues are methylated [87]. These methylated lysine residues apparently have an immunological significance and comprise T-cell epitopes in heparin-binding hemagglutinin [113]. There is ample evidence that many post-translational modifications are significant for the immune system and for protecting the organism against tuberculosis.

The lifetime of cellular proteins, their interaction with other proteins, and enzymatic activity are regulated via PTMs. The PTMs of many eukaryotic proteins comprise the necessary stage of protein maturation.

Proteins not subjected to PTMs turn out to be functionally inactive [114]. The role of the PTMs of mycobacterial proteins still remains poorly understood.

**Proteogenomic analysis**

All the studies we have described show that the genomic variability of mycobacterial strains is reflected at the proteomic level; hence, the data of comparative proteomics may be helpful in understanding the phenotypic differences of different groups of bacteria, such as the degree of drug sensitivity and virulence. On the other hand, proteomic studies facilitate the correct deciphering of genomic information.

**Fig. 3:**
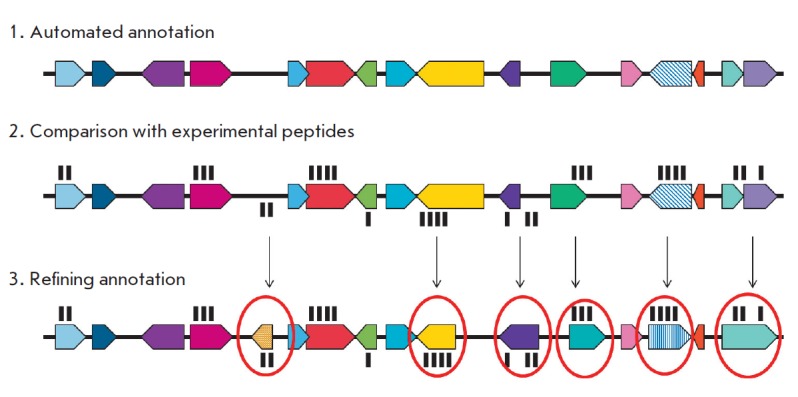
Refining annotation using mass spectrometry data

Most mass spectrometric techniques rely on databases containing annotated amino acid sequences of the protein. However, a comparative analysis has shown that annotation based on genomic data is often incomplete and contains errors. For example, the genomic sequence of the *M. tuberculosis *strain H37Rv was fully deciphered at the Sanger Institute in 1998 [43] and shown to contain 3,924 open-reading frames (ORF). However, a few years later, the authors reported an increase in ORF to 3,995 [115]. The annotated version of *M. tuberculosis *H37Rv (the 27th version according to the TubercuList database) currently contains 4,018 protein-coding genes, 26% of which belong to the class of proteins with a hypothetical function. Moreover, proteomic studies have largely facilitated the processing of genomic annotation by presenting experimental evidence for a series of genes that had not been previously annotated or genes whose transcription initiation sites had been incorrectly identified, as well as by simply confirming the existing ORF (*Fig. 3*). Kelkar et al. identified 3,176 proteins of *M. tuberculosis *H37Rv and 250 peptides not corresponding to the existing annotation based on the data of a MS/MS analysis in 2011. As a result, the annotation was supplemented with 41 proteins and the transcription initiation sites of 33 genes were specified [34]. The same year, Norwegian researchers refined the annotation of 24 genes of the H37Rv strain using the MS approaches [116]. In general, several large proteogenomic studies of *M. tuberculosis *have been conducted over the past few years, where each of the studies described between 20 and 40 unannotated proteins, and existing genome annotations have been refined [23, 34, 116, 117].

## CONCLUSION

The advances in proteomics have opened up new approaches in studying tuberculosis by making it easier to find solutions to many complex problems, including the interactions between bacteria and the host cell. Despite the fact that proteomics lags behind genomics and transcriptomics due to limitations in instruments and insufficient sensitivity, an increasing number of studies involving proteomic approaches for the investigation of infectious agents are being published. For example, virulence factors and their mechanisms of action, host and pathogen response to the infectious process have been described using a proteomic analysis. Proteomics has made it possible to describe the unique features of various *M. tuberculosis *strains more thoroughly.

Studying the tuberculosis pathogen at the proteomic level can contribute to the identification of the metabolic and physiological characteristics necessary for a successful course of infection, as well as the virulence mechanisms that allow *M. tuberculosis *to modulate the host’s immune response. The proteins synthesized during the entry of mycobacteria into the host’s cells are important for their survival under these conditions: so they are considered as potential targets for developed drugs. Designing new drugs and treatment regimens is especially topical today, when strains of multiple types and extensive drug resistance continue to spread. Hence, studying the complete proteomic profile of mycobacteria may contribute to a better understanding of pathogen physiology and even tuberculosis treatment.

## Abbreviations

MS: mass-spectrometry
HPLC: high-performance liquid chromatography
MRM: multiple reaction monitoring
PTM: post-translational modifications
ORF: open reading frame
TB: tuberculosis
